# 

**Published:** 1865-09

**Authors:** 


### CONTENTS.

### ORIGINAL CONTRIBUTIONS.

ART. XXVIII. CASES IN PRACTICE. By WALTER HAY, M.D., Chicago, 545

ART. XXIX. PUERPERAL FEVER, COMPLICATED WITH SECONDARY UTERINE HEMORRHAGE. By SWAYNE WICKERSHAM, M.D., Chicago, ----- 549

ART. XXX. SYNOPSIS OF THE ETIOLOGY AND TREATMENT OF TYPHOID FEVER. By ROSS C. RUSS, M.D., Royalton, Ind., ----- 555

ART. XXXI. BEE BREAD AS A DIURETIC. By JAMES S. WHITMIRE, M.D., Metamora, Ill., ----- 557

ART. XXXII. REPORT ON THE SANITARY CONDITION OF CHICAGO, AND PREVALENCE OF DISEASES, FROM APRIL 1, TO AUGUST 1, 1865. By N. S. DAVIS, M.D. AND PROF., Chicago, ----- 559

### PROCEEDINGS OF SOCIETIES.

Proceedings of the DeWitt County Medical Society, ----- 566

### BOOK NOTICES.

The Renewel of Life. Lectures chiefly Clinical. By Thos. King Chambers, M.D., Honorary Physician to the Prince of Wales, &c., ----- 567

The Army Surgeon's Manual. By William Grace, of Washington, D.C., - 568

Physician's Prescription Book. By Jonathan Perira, M.D., F.R.S., ----- 569

### EDITORIAL.

Medical Schools in Chicago, ----- 569

Deaths:— -----

Professor D. L. McGugin, of Keokuk, ----- 570

Dr. James Couper, of New Castle, Delaware, ----- 571

Dr. Melvin N. Rust, Surgeon of U.S. Vols., ----- 572

American Dental Association, ----- 573

Address of Dr. Allport, ----- 573

Remarks of Dr. N. S. Davis to the Members of the American Dental Association, ----- 576

On the Diagnosis and Treatment of Scabies, ----- 583

Transactions of Illinois State Medical Society, ----- 585

American Medical Association, ----- 586

City Mortality for June and July, 1865, ----- 586

Medical Practice Wanted, ----- 587

Transactions of the American Medical Association, ----- 588

Antiseptic Injection for the Cadaver, ----- 588

Anæsthesia by Electricity, ----- 589

Premium Offered, ----- 589

